# Fukushima Nuclear Accident Recorded in Tibetan Plateau Snow Pits

**DOI:** 10.1371/journal.pone.0116580

**Published:** 2015-02-06

**Authors:** Ninglian Wang, Xiaobo Wu, Natalie Kehrwald, Zhen Li, Quanlian Li, Xi Jiang, Jianchen Pu

**Affiliations:** 1 State Key Laboratory of Cryospheric Science, Cold and Arid Regions Environmental and Engineering Research Institute, Chinese Academy of Sciences, Lanzhou, China; 2 CAS Center for Excellence in Tibetan Plateau Earth Sciences, Beijing, China; 3 Department of Environmental Science, Informatics and Statistics, University of Venice, Venice, Italy; 4 College of Atmospheric Science, Nanjing University of Information Science & Technology, Nanjing, China; Institute of Tibetan Plateau Research, CHINA

## Abstract

The β radioactivity of snow-pit samples collected in the spring of 2011 on four Tibetan Plateau glaciers demonstrate a remarkable peak in each snow pit profile, with peaks about ten to tens of times higher than background levels. The timing of these peaks suggests that the high radioactivity resulted from the Fukushima nuclear accident that occurred on March 11, 2011 in eastern Japan. Fallout monitoring studies demonstrate that this radioactive material was transported by the westerlies across the middle latitudes of the Northern Hemisphere. The depth of the peak β radioactivity in each snow pit compared with observational precipitation records, suggests that the radioactive fallout reached the Tibetan Plateau and was deposited on glacier surfaces in late March 2011, or approximately 20 days after the nuclear accident. The radioactive fallout existed in the atmosphere over the Tibetan Plateau for about one month.

## Introduction

On March 11, 2011, a magnitude 9.0 earthquake occurred on the sea floor approximately 130 km east of Port of Sendai, Honshu Island, Japan, and triggered a 10 m high tsunami, causing tremendous devastation along the east coast of Japan. The power supply to the nuclear power plant at Fukushima (37.42°N, 141.03°E) was affected by the earthquake and tsunami, resulting in the shutdown of the cooling, thereby causing several nuclear reactors to explode and releasing large amount of radioactive nuclear substances (radioactive fallout) to the atmosphere. The freshwater and seawater used to cool the nuclear reactors were highly polluted by the radioactive nuclear substances before flowing back to the ocean. The released radioactive nuclear substances not only polluted Japanese soil and coastal seawater [1, 2], but also spread to other areas of the Northern Hemisphere via atmospheric circulation and ocean currents [3, 4], affecting the hemispheric and even global environment. As an overdose of nuclear radiation may seriously threaten human health [5] and wildlife survival [6, 7], this nuclear accident has caught the attention of the world.

The radioactive fallout released by the Fukushima power plant has been detected in the atmosphere, soil, surface water, and pastures in the low-altitude regions of North America and Eurasia [8–12]. Much of the radioactive material was transported by the westerlies, resulting in the fallout over North America. However, in order to reach the Tibetan Plateau, material transported by the westerlies has to first circle much of the globe. The wet and dry deposition of this radioactive fallout also dilutes the atmospheric concentrations during long-distance transport. The aim of this study is to investigate if the Fukushima radioactive fallout could be detected on the remote high-altitude Tibetan Plateau, which is mostly controlled by the westerlies during the period from the autumn to the next spring, using an array of snow pits across the Tibetan Plateau. The Fukushima nuclear accident released large amount of radioactive ^137^Cs which can generate β rays during its decay. We analyzed the β radioactivity in Tibetan Plateau snow pits to determine if the nuclear accident affected the Tibetan Plateau, and if so, when the released radioactive fallout arrived in this area. This timing of the arrival of the radioactive fallout can help verify the simulated pollutant diffusion by atmospheric dispersion models.

## Sampling and Analytical Methods

**Figure 1:**
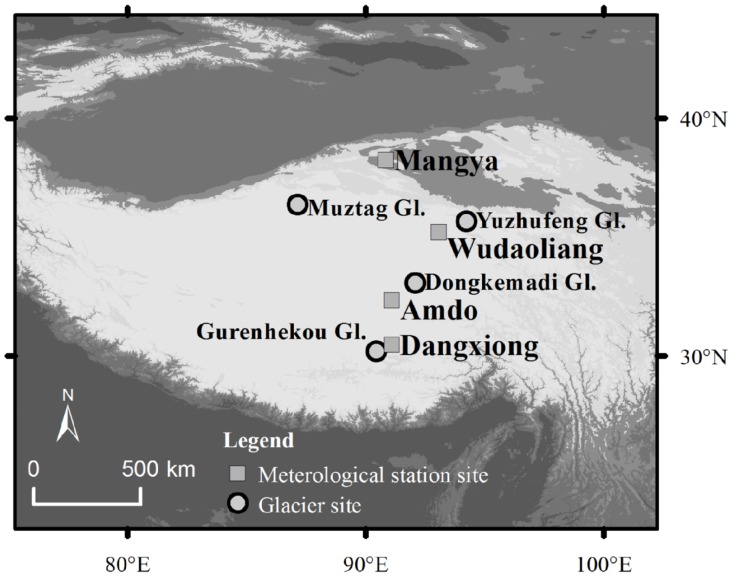
Locations of the study glaciers and their adjacent meteorological stations on the Tibetan Plateau.

Our study sites are located on the four glaciers in the Tibetan Plateau (Fig. 1); no specific permissions are required for these sites. Our field works were just sampling snow, did not involve endangered or protected species. The longitudes and latitudes of our four study sites are listed in Table 1.

**Table 1:** Locations of snow pits and sampling dates on the different Tibetan Plateau glaciers.

**Glacier**	**ELA (m, 2008/09)**	**Snow pit location**		**Snow thickness (cm)**	**Sampling date**
		**Altitude (m)**	**Latitude and Longitude**		
Yuzhufeng Gl.	5400	5478	35°39′08.9″N 94°13′55.2″E	60	2011.05.06
Dongkemadi Gl.	5650	5642	33°04′13.0″N 92°04′59.6″E	55	2011.05.11
Gurenhekou Gl.	5810	5720	30°11′26.5″N 90°27′05.0″E	40	2011.05.15
Muztag Gl.	5650	5708	36°21′41.4″N 87°08′45.1″E	40	2011.05.31

**Figure 2:**
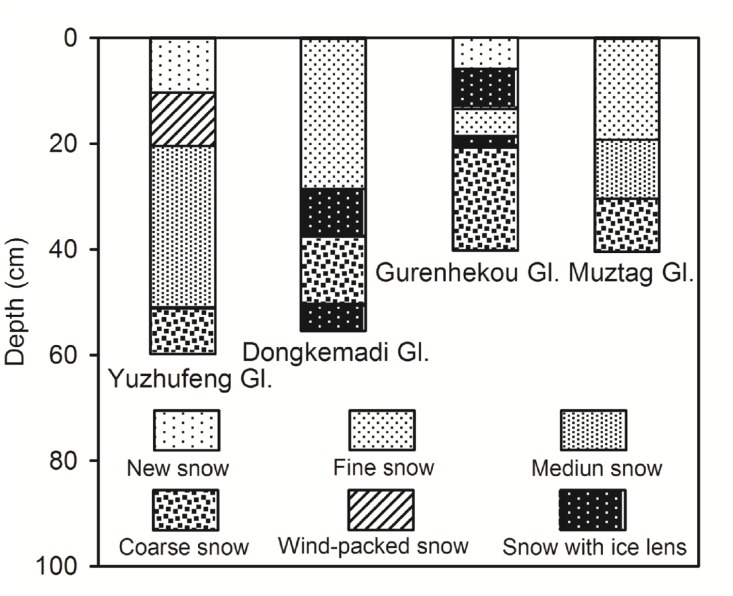
Stratigraphic profiles of the snow pits on the Tibetan Plateau study glaciers in spring 2011. The very thin ice layers in snow pits (snow with ice lens) were formed by the refreezing of the small melt on the snow surface caused by the solar radiations in the cold accumulation period.

In May 2011, snow-pit samples were collected on the Gurenhekou Glacier in the Nyainqentanglha Range, Dongkemadi Glacier in Tanggula Mountains, and Muztag Glacier and Yuzhufeng Glacier in the Kunlun Mountains in the Tibetan Plateau (Fig. 1). Each snow pit was located near the equilibrium line altitude (ELA) of its glacier (see Table 1), and was dug to the glacial ice surface, in order to ensure that the collected snow samples contained the snow that had fallen since the autumn of 2010. The stratigraphic characteristics of each snow pit are illustrated in Fig. 2.

When collecting samples, the snow on each snow-pit wall surface was first removed using a clean plastic knife in order to minimize mixing snow at different depths during the course of digging; Next, 150 mL wide mouth polyethylene plastic bottles, which were pre-cleaned using deionized water in laboratory, were horizontally inserted into the snow layers to collect samples. The sampling interval in each snow pit was approximately 10 cm, and 10 parallel samples in the same snow layer were collected (in which, 9 samples were mixed together and utilized for the analysis of the β radioactivity, and the left 1 sample for δ^18^O analysis). All samples were transported in a frozen state to the State Key Laboratory of Cryospheric Science (SKLCS) in Lanzhou for analyses and were analyzed immediately after arriving at the laboratory.

In a Class 100 clean room of the SKLCS, each β radioactivity sample (about 0.7~1.0 kg in weight) was first melted at room temperature, and then spiked with 4 mol/L HCl until reaching a pH value of 2 in order to activate radioactive substances. Next, the sample solution was filtered 3 times through MN616LSA-50 cation and MN616LSB-50 type anion membranes, so that the radioactive substances were completely absorbed by the membranes. The membranes were then placed on tinfoil and dried at room temperature. A Mini 20 Alpha-Beta Multidetector (Eurisys Mesures Company) ran idly for 72 hours in order to reach a stable state, and then we measured the background β radioactivity, only about 0.21±0.04 cpm (counts per minute). All prepared samples were measured for 24 hours each using the Alpha-Beta Multidetector. The each sample’s β radioactivity (dph/kg, disintegrations per hour in one kilogram sample) was calculated by deducting its background value from its measured value.

We analyzed δ^18^O in snow-pit samples at the SKLCS using a Picarro L1102-i Liquid Water Isotope Analyzer with a measurement accuracy of <0.1‰. Due to the seasonal variations in δ^18^O in precipitation over the Tibetan Plateau, the δ^18^O profile of the each snow pit provides information regarding the period during which the snow of each pit was accumulated. We estimated the relative timing of the fallout of radioactive material from the Fukushima incident over the Tibetan Plateau based on the δ^18^O profile and the depth of the peak β radioactivity in the each snow pit.

## Results and Discussion

**Figure 3:**
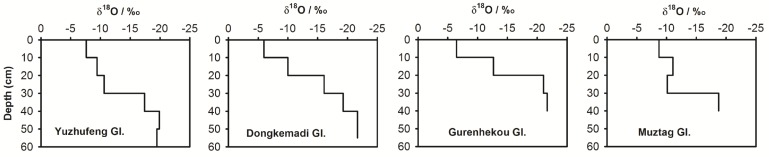
Variations of δ^18^O with depth in the snow pits on the study glaciers in the Tibetan Plateau.

Previous investigations demonstrate that the seasonal variations of δ^18^O in precipitation and ice cores in the northern Tibetan Plateau are controlled mostly by the changes in air temperature, with high δ^18^O in the summertime and low δ^18^O in the wintertime [13–15]; However, in the southern Tibetan Plateau, the amount effect results in lower δ^18^O in precipitation and ice cores in the summertime [15–17]. A recent study indicates that δ^18^O in precipitation in Naqu and Lhasa (where the Gurenhekou Glacier is located between these two sites) is higher in the springtime than in winter and autumn [15]. These trends imply that the general decrease of δ^18^O with depth in the four snow pits (Fig. 3, see also S1 Dataset) reflect that the snow in these pits accumulated during the period from the previous autumn to the sampling time, i.e., from the autumn of 2010 to the spring of 2011.

**Figure 4:**
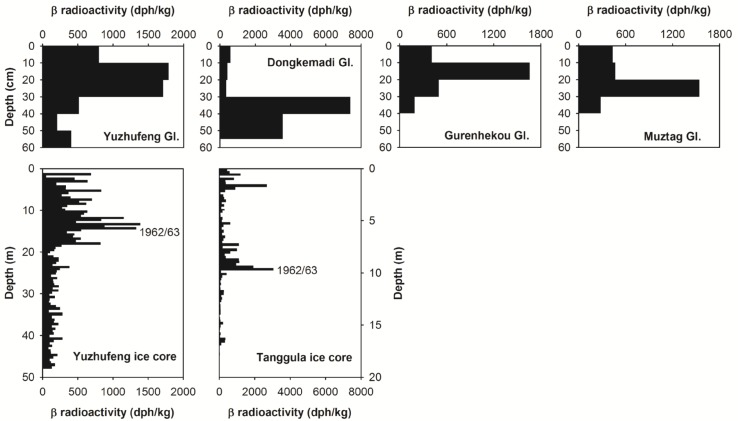
Profiles of the β radioactivities recorded in the four study snow pits and two Tibetan Plateau ice cores.

The peak β radioactivity in each snow pit profile (Fig. 4, see also S1 Dataset) is crucial evidence that the Fukushima radioactive fallout travelled from Japan spread to the Tibetan Plateau. Each snow pit demonstrates a prominent β radioactivity peak, with values ranging of 7391 dph/kg (Dongkemadi Glacier), 1786 dph/kg (Yuzhufeng Glacier), 1657 dph/kg (Gurenhekou Glacier), and 1541 dph/kg (Muztag Glacier) respectively. These values are 21.5, 8.5, 8.8, and 5.5 times larger than the minima in their corresponding snow pits, i.e., 343 dph/kg, 208 dph/kg, 189 dph/kg, and 281 dph/kg, respectively.

We investigate if these peak β radioactivities are related to the seasonal variations of β radioactivity in snow layers on Tibetan Plateau glaciers, which may be caused by the seasonal variations of net accumulation rate and/or dust concentration, or if we can definitively ascribe the β radioactivity peaks to the deposition of radioactive Fukushima fallout. If these peaks exceed the β radioactivity peaks in their corresponding local snow or ice core records over a longer period (such as over past 30 years) and there are no correlations between the β radioactivity and dust concentration in snow and ice in the Tibetan Plateau, the influence of seasonal variations in net accumulation rate and dust concentration to create these peaks should be easily eliminated, and thus the peak β radioactivities in the study snow pits can likely be ascribed to the Fukushima nuclear accident.

**Figure 5:**
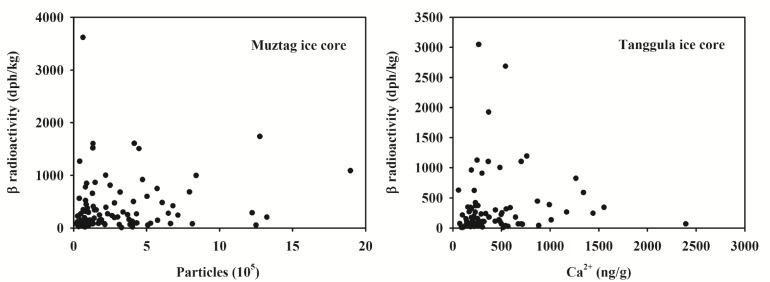
Correlations between the β radioactivity and dust concentration in the Muztag and Tanggula ice cores from the Tibetan Plateau.

In 2005 and 2007, we drilled ice cores on the Longxiazailongba Glacier (adjacent to the Dongkemadi Glacier) in the Tanggula Mountains (Tanggula ice core) and the Yuzhufeng Glacier in Kunlun Mountains (Yuzhufeng ice core). The β radioactivity records in these two ice cores are presented in Fig. 4 (see also S2 Dataset). Clearly, the peak β radioactivities in the snow pits in the Yuzhufeng Glacier and Dongkemadi Glacier are much higher than that in the corresponding local ice cores, and even overwhelm the peak β radioactivities caused by past atmospheric thermonuclear tests in the early 1960s. The Fig. 5 illustrates the correlations between the β radioactivity and dust concentration in the Muztag and Tanggula ice cores (Ca^2+^ concentration is a proxy of dust content). It obviously shows that there are no correlations between them, which imply that the variations of the β radioactivity in the Tibetan snow and ice are not directly connected with dust. All these suggest that the peak β radioactivities in the study snow pits were produced by the Fukushima nuclear accident.

If we regard the β radioactivity in ice cores prior to 1950s (corresponding to depths lower than 20 m for the Yuzhufeng ice core, and lower than 12 m for the Tanggula ice core) as the background levels, i.e., 74−382 dph/kg (with a mean of 163 dph/kg) for the Yuzhufeng ice core and 7−346 dph/kg (with a mean of 80 dph/kg) for the Tanggula ice core, then the minimum snow pit β radioactivities on the Yuzhufeng Glacier and Dongkemadi Glacier are in the range of their local respective background levels. The peak β radioactivities for the Yuzhufeng Glacier and Dongkemadi Glacier are 11.0 and 92.4 times larger than their local average background levels, respectively.

**Figure 6:**
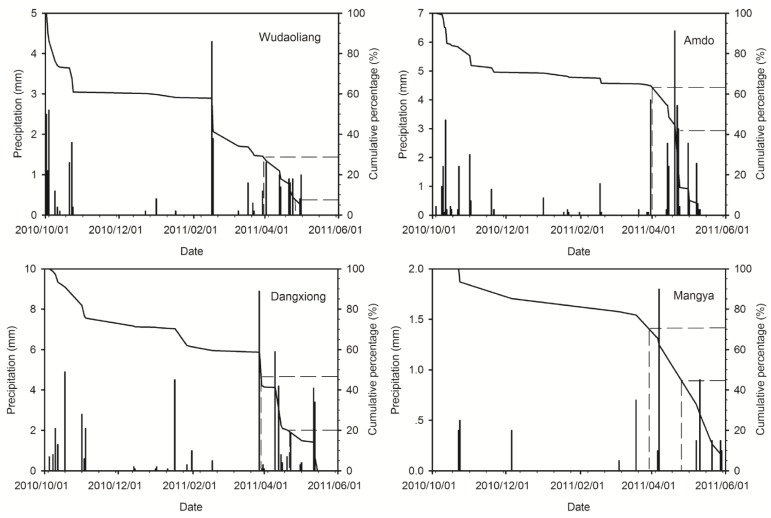
Daily precipitation variations and their cumulative percentages at different meteorological stations close to the study glaciers in the Tibetan Plateau from October 1, 2010 to the sampling dates in May 2011. Thin columns stand for daily precipitations while solid curves for their cumulative percentages calculated backward. Horizontal dashed lines represent the ratios of net accumulation amounts above the depths of the top and bottom limits of snow layer with the peak β radioactivity to the total net accumulation amount in the each study snow pit. The vertical dashed lines demonstrate the dates that the horizontal dashed lines intersect the curve of precipitation cumulative percentage. The dates determined using this technique correspond to the starting and ending dates of the deposition of Fukushima fallout on the surfaces of the Tibetan Plateau glaciers.

The peak β radioactivities appear at different depths in different snow pits (Fig. 4). We investigate if these different depths suggest that the Fukushima radioactive fallout was deposited on different areas of the Tibetan Plateau during different time periods and/or if wet versus dry deposition affected the timing of fallout. We examined the daily variations of precipitation at the study snow pits during the time periods corresponding to snow accumulation in the pits. Due to the lack of ongoing precipitation observations on the study glaciers, we used precipitation data from the closest meteorological stations to these glaciers (see Fig. 1, Dangxiong Station for Gurenhekou Glacier, Amdo Station for Dongkemadi Glacier, Wudaoliang Station for Yuzhufeng Glacier, and Mangya Station for Muztag Glacier). The daily precipitation variations at the different stations demonstrate that substantially more precipitation fell at Amdo and Mangya Stations after the middle of March 2011 than at the other two stations (Fig. 6). This increased precipitation explains why the peak β radioactivities were located at deeper depths in the snow pits on the Dongkemadi Glacier and Muztag Glacier than in the other two snow pits. Considering that the accumulation season usually begins at the beginning of October for the Tibetan Plateau glaciers, and since the the snow pits are all located near their respective ELAs (in fact, during 1 to 8 November 2010, we measured mass balance sticks on these study glaciers, and found that there were only about 10–20 cm fresh snow and no firn near the sampling sites), we assume that the snow in all of the snow pits accumulated during between October 2010 to the sampling dates in May 2011. Therefore, we estimate the relative age of the snow at different depths by calculating the ratio of the net accumulation above a certain depth to the total net accumulation amount in the same snow pit (in short, the net accumulation ratio). The densities of the different types of snow are required in order to compute the net accumulation amount. We determined the densities of new snow, fine snow, medium snow, coarse snow, wind-packed snow and snow with ice lens during our field surveys as 0.14, 0.25, 0.30, 0.40, 0.35, and 0.55 g/cm^3^, respectively, which correspond with previously published data [18]. By comparing the net accumulation ratio with the cumulative precipitation percentage at the corresponding station (Fig. 6), which was calculated starting from the sampling date backward to October 1, 2010, we could estimate the timing of the Fukushima radioactive fallout deposited on the Tibetan Plateau glaciers.

**Figure 7:**
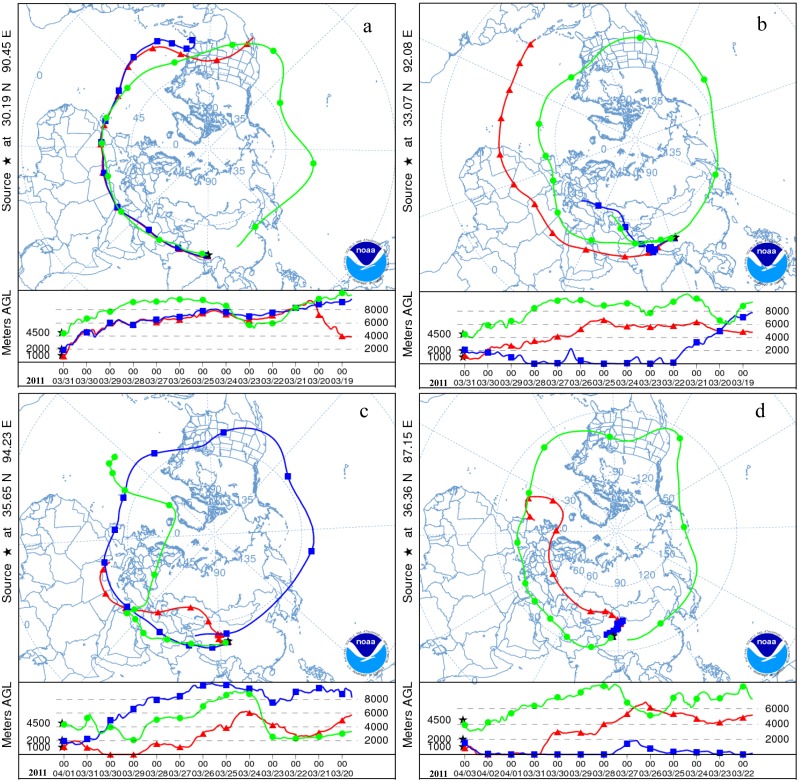
The backward air parcel trajectories at the heights of 1000 m, 2000 m and 4500 m above ground level of the study sites at about the starting dates of the Fukushima fallout deposition on the Tibetan Plateau glaciers. They were computed by using the HYSPLIT model and the GDAS meteorological data (http://www.arl.noaa.gov/HYSPLIT.php). Panel (a) represents the status as for the Gurenhekou Glacier, (b) for the Dongkemadi Glacier, (c) for the Yuzhufeng Glacier, and (d) for the Muztag Glacier.

The resulting estimated times for the snow layers with peak β radioactivities attributed to the Fukushima radioactive fallout are illustrated in Fig. 6 and listed in Table 2. These dates show that the Fukushima fallout deposited on the Tibetan Plateau glaciers occurred during nearly the same time period, i.e., from approximately the end of March 2011 to the late April 2011. This timing suggests that it took about 20 days for the Fukushima radioactive fallout to be transported to the Tibetan Plateau via the westerlies, and the radioactive fallout existed in the atmosphere over the Tibetan Plateau for about one month. This timing is consistent with fallout monitoring observations, which demonstrate that the Fukushima radioactive nuclear substances arrived in the US on March 15, 2011 via atmospheric circulation with peak concentrations appearing on March 23, 2011 [11], arrived in Europe on March 20, 2011 [9] with peak concentrations appearing during April 4–6, 2011 [19–21] and with no detected fallout after April 28, 2011 [21]. A recent study indicates that the hemispheric transport of the Fukushima radioactive fallout by the westerlies took approximately 18 days [22]. The Fig. 7 displays the backward air parcel trajectories at the heights of 1000 m, 2000 m and 4500 m above ground level of the study sites at about the start time of the Fukushima fallout deposition, which were computed by using the HYSPLIT model and the GDAS meteorological data (http://www.arl.noaa.gov/HYSPLIT.php). It clearly indicates that the westerlies in the upper troposphere of the northern hemisphere takes about 10 to 14 days to turn a circle around the Earth while the air in the lower troposphere moves slowly relatively, and the air in the lower and upper troposphere can exchange and mix during its movements, and the air over the Japan can be transported to the Tibetan Plateau by the westerlies. Moreover, it is also revealed that the air in the middle or lower troposphere in the northern mid-latitudes can take 10 to 12 day to travel nearly a semicircle around the Earth. Those not only demonstrate the Fukushima radioactive fallout could be transported to the Tibetan Plateau by the westerlies, but also support the above result of the timing of the Fukushima radioactive fallout reached to and deposited on the Tibetan Plateau glaciers estimated by comparing the snow accumulations and the positions of the peak β radioactivities in the study snow pits.

**Table 2:** Time period of Japan Fukushima fallout deposited on the glaciers in the Tibetan Plateau estimated by the positions of the peak β radioactivaties in the study snow pits.

**Glacier**	**Net accumulation ratio since the start of fallout deposition (%)**	Deposition start time*	**Net accumulation ratio since the end of fallout deposition (%)**	Deposition end time*
Yuzhufeng Gl.	27.4	2011.03.31 (03.30–04.02)	8.0	2011.04.25 (04.24–04.30)
Dongkemadi Gl.	64.1	2011.03.31 (03.29–03.31)	42.2	2011.04.21 (04.20–04.22)
Gurenhekou Gl.	46.8	2011.03.29 (03.28–03.30)	20.2	2011.04.20 (04.20–04.23)
Muztag Gl.	71.4	2011.03.28 (03.19–04.06)	44.6	2011.04.22 (04.07–05.08)

* The date (year.month.day) outside of the parenthesis is the optimal estimate time by using the net accumulation ratio along with the cumulative precipitation percentage curve. The dates (month.day-month.day) in the parentheses are the possible time period estimated by the net accumulation ratio along with the two adjacent cumulative precipitation percentages, and the large time span estimated on the Muztag Glacier might be resulted from the application of the meteorological data from the Mangya Station which is far from the glacier (there is no other stations closer to the glacier than the Mangya Station).

It’s noted that the peak value of the β radioactivity in the snow pit on the Dongkemadi Glacier was much higher than the others (see Fig. 4). We calculated the averages of the β radioactivities in the four study snow pits by the accumulation-weight method, and found that the averages were 3154 dph/kg (Dongkemadi Glacier), 911 dph/kg (Yuzhufeng Glacier), 706 dph/kg (Gurenhekou Glacier) and 638 dph/kg (Muztag Glacier) respectively. The accumulation-weight average of the β radioactivities was still larger in the snow pit on the Dongkemadi Glacier. However, if removed the peak β radioactivities, the accumulation-weight averages were 437 dph/kg (Dongkemadi Glacier), 432 dph/kg (Yuzhufeng Glacier), 362 dph/kg (Gurenhekou Glacier), and 385 dph/kg (Muztag Glacier) respectively, which are at the comparable level. These imply that there was much more fallout deposited on the Dongkemadi Glacier which is located in the middle of the Tibetan Plateau. This might be related to the situations of atmospheric circulation during the fallout deposition period and/or other reasons, and need to be further investigated.

The Fukushima nuclear accident, like the 1986 Chernobyl nuclear accident [23, 24], created a radioactive horizon that can be used as independent age markers in snow and ice cores in the Northern Hemisphere. And more importantly, the presence of these radioactive horizons in snow and ice cores extending back to the early 1950s with thermonuclear tests in the South Pacific demonstrate that humans have been injecting radioactive material into both the atmosphere and hydrosphere for decades with hemispheric to global impacts [25–28]. Ice cores directly trap and archive this radioactive fallout, and also provide a natural background level of β radioactivity before any thermonuclear tests or reactor accidents, providing a direct record of the impacts of human activities on the Earth’s environment.

## Conclusions

The 2011 Fukushima nuclear accident in Japan and its environmental impact have drawn much attention across the globe. Worldwide monitoring and research on the dispersion and transport of the released radioactive fallout by this nuclear accident have been conducted mostly in low-altitude regions, and scarce information exists in high-altitude regions. In this study, we analyzed the β radioactivity in the snow pits containing snow encompassing the autumn of 2010 to the spring of 2011 on four Tibetan Plateau glaciers and determined a β radioactivity peak caused by the Fukushima nuclear accident in each of the four snow-pit profiles. This β radioactivity peak creates a new reference layer in glacier snow and ice, which can be used in the future ice core chronologies as an independent dating method. This study also reveals that the radioactive fallout released by the Fukushima nuclear accident spread to the Tibetan Plateau by the westerlies in approximately 20 days after incident. This result suggests that atmospheric pollutants may be dispersed across a hemisphere via tropospheric circulation in approximately 20 days.

## Supporting Information

S1 Dataset The β radioactivities and δ^18^O recorded in the Tibetan snow pits. (XLS)

S2 Dataset The β radioactivities recorded in the Tanggula and Yuzhufeng ice cores from the Tibetan Plateau. (XLS)
